# Tomato Root Exudates Infected by *Meloidogyne incognita* Impact the Colonization of Nematicidal *Proteus vulgaris*

**DOI:** 10.3390/microorganisms12112188

**Published:** 2024-10-30

**Authors:** Pengyan Yue, Qianru Hu, Wenzhen Zhou, Xiaozhan Rang, Yajun Liu

**Affiliations:** State Laboratory for Conservation and Utilization of Bio-Resources, Yunnan University, Kunming 650091, China; yuepengyan@stu.ynu.edu.cn (P.Y.); hqr910954216@163.com (Q.H.); 12021114082@mail.ynu.edu.cn (W.Z.); rangxiaozhan@itc.ynu.edu.cn (X.R.)

**Keywords:** tomato exudates, root-knot nematodes, chemotaxis, biocontrol effect

## Abstract

Root exudates play a pivotal role in shaping the microbial community in the rhizosphere and can impact the efficacy of bacteria in controlling nematode populations. This study identified *Proteus vulgaris* BX-1 as significantly effective in controlling *Meloidogyne incognita*. The infection of tomato plants with this nematode induced noticeable alterations in the composition of tomato root exudates and led to an increased colonization rate of strain BX-1. Further investigation into how strain BX-1 responded to changes in tomato root exudates revealed that specific metabolites, such as caffeic acid, coumarin, salicylic acid, sebacic acid, and butyric acid, strongly attracted strain BX-1. This attraction potentially contributed to its enhanced colonization and improved efficiency in controlling nematodes. Understanding the correlation between specific metabolites in root exudates and the response of antagonistic bacteria provides valuable insights for enhancing their effectiveness as biological control agents against plant-parasitic nematodes.

## 1. Introduction

Root-knot nematodes (*Meloidogyne* spp.) are widely distributed and highly destructive plant parasitic nematodes [1], causing an estimated 78 billion USD in crop losses annually [2]. Chemical nematicides have been the most effective method for controlling root knot nematodes but their high toxicity, environmental pollution, and development of resistance have led to their prohibition [3,4]. Therefore, there is a critical need to identify novel, safe, and efficient alternatives to chemical nematicides. Biological control using fermentation broth and secondary metabolites from microorganisms may offer a promising alternative [5].

Plant root secretions play a crucial role in supporting plant development and interactions with physical and biological factors in the rhizosphere. During stress conditions, root exudates help to enhance plant resource utilization efficiency and facilitate interactions between plants and soil microorganisms, thereby improving stress tolerance [6]. Numerous studies have shown that amino acids, small-molecule sugars, aromatic compounds, and small-molecule organic acids are important for promoting the colonization and chemotaxis of inter-root bacteria [7]. de Weert [8] reported that malic and citric acids are essential for the colonization and chemotaxis of *Pseudomonas fluorescent* WCS365 in the tomato root system. When invaded by *Fusarium oxyspora* P1 L43 in the presence of the *P. fluorescens* biocontrol strain WCS365, there was a significant increase in total organic acid content, primarily due to a strong increase in citric acid levels. Additionally, succinic acid levels decreased dramatically [8,9]. Liu et al. [10] observed that cucumber roots colonized by *F. oxysporum* f. sp. *cucumerinum* or SQR9 increased tryptophan secretion to further strengthen SQR9 colonization. In contrast, colonized cucumber roots reduced raffinose secretion to inhibit *F. oxysporum* f. sp. *cucumerinum* colonization [11]. Ray et al. [12] identified shikimic acid, gallic acid, fumaric acid, ferulic acid, vanillic acid, and quercetin as the key components in okra root secretions that contribute to the endogenous *Bacillus faecalis* BHU 12, BHU 16, and BHU M7. Huang et al. [13] observed a significant increase in jasmone acid levels in *Arabidopsis thaliana* (L.) Heynh infected with the pathogenic bacterium *P. syringe*. They also noted a significant increase in amino acids, nucleotides, and long-chain organic acids (LCOAs), while sugar content was low. Nucleotides and long-chain organic acids play a crucial role in recruiting beneficial microbes. Shu et al. [14] reported that 3-carene effectively inhibits *P. fluorescens*. Yang et al. [15] found that *Bacillus vileness* LG14-3 exhibits chemotaxis towards banana root (*Musa acuminata* Cavendish cv. Brail) secretions, while Gao et al. [16] observed that *B. amyloliquefaciens* TR2 promotes watermelon (*Citrullus lanatus*, Meijiao No. 4, China) plant growth, and its root secretions enhance bacterial colonization.

*Proteus* spp. bacteria were described in 1885 by Gustav Hauser, who observed their characteristic of vigorous swarming growth [17]. *Proteus* spp., despite being a human enteric pathogen, are widely distributed in soil and water and possess various functions such as urea decomposition [18], heavy metal removal [19], and biodegradation of pesticides and antibiotics [20], among others. The nematicidal activity of *Proteus* spp. penner was first noted in 2014 [21]. In this research, the biocontrol efficacy of *Proteus vulgaris* BX-1 was assessed. The colonization rate of strain BX-1 was observed to be influenced by *M. incognita* infestation. Furthermore, alterations in tomato root secretion and chemotaxis of the antagonistic strain BX-1 were further investigated to comprehend the role of this response.

## 2. Materials and Methods

### 2.1. Preparation of Nematodes and Experimental Strains

Egg masses from *M. incognita* were isolated from tomato (*Solanum lycopersicum* L.) root knots grown in greenhouse pots. *P. vulgaris* BX-1 was isolated from rhizosphere soil infected by *M. incognita* in the Renhe tobacco field in Panzhihua, Sichuan, China, stored in the State Key Laboratory of Conservation and Utilization of Yunnan Biological Resources, and BX-1 was cultured in the LB liquid [22] medium for 16 h with incubation at 37 °C. The strain *Proteus vulgaris* underwent 16S rRNA sequencing, indole, ornithine decarboxylase, and H_2_S tests [23].

### 2.2. Nematicidal Activity Test

The fermentation broth of *P. vulgaris* BX-1 (5 mL, OD600 = 1.50) was centrifuged at 5000 RMP for 5 min, and the remaining broth was extracted three times with ethyl acetate. The organic phase was concentrated, evaporated, weighed, and prepared at final concentrations of 2 mg/mL and 1 mg/mL in a 5% methanol solution. Subsequently, 200 J2 were gently added and stirred with 1 mL sample in a 3 cm cell culture dish. Each strain was treated with sterile water as control and a sterile LB liquid medium with three parallel and three replicates, respectively. The culture dish was placed in a dark incubator at 28 °C, and the total number of nematodes and the number of deaths were recorded under a microscope at 4 h, 8 h, 12 h, 24 h, and 48 h, respectively. The mortality was then calculated [24].

### 2.3. Green Fluorescent Labeling of Proteus vulgaris BX-1

A single colony of *P. vulgaris* was selected and transferred to a liquid LB test tube and then cultured overnight at 37 °C. After transferring 1% to a liquid LB medium, the initial OD600 was measured, and the culture was continuously oscillated with additional measurements taken every 0.5 h to plot the growth curve. The following day, 1% of the culture was transferred to 50 mL of LB medium, and organisms were collected when the growth reached OD600 = 0.8 (approximately 2.5 h). The cells were then washed three times with 10% glycerol and resuspended in 2 mL of 10% glycerol to create sensory cells. Each tube was filled with 90 μL of plasmid for transformation, including a total of 35 μL of green fluorescent plasmid (plasmid: sfgsp-MCS). The sensory cells were incubated on ice for 5 min, electrically transformed at 2500 KV, coated with a resistance plate, and incubated overnight at 37 °C. A single clone was picked into 10 μL of sterile water and mixed; then, 0.5 μL was used as a template for colony PCR amplification. The products were detected by gel electrophoresis. Positive transformants were selected, smeared, and observed under a blue excitation light to determine the presence of green fluorescence. The plasmid stability of the labeled strains was determined according to a previous study to verify their nematicidal rate and growth.

### 2.4. Pot Experiment

Four treatments were established in the experiment, with five replicates for each treatment—T1: blank control (sterile water); T2: inoculation with 2000 J2 of *M. incognita* after three days; T3: inoculation with 30 mL of *P. vulgaris* BX-1 fermentation broth at OD600 = 1.5 (108 cfu/mL) and 2000 *M. incognita* J2 after seven days; T4: inoculation with fermentation broth. Tomato seedlings were transplanted into polypropylene cups (8.5 cm diameter and 10 cm height) in a plastic greenhouse (26–28 °C, soil pH 5.5–6.0). Galls and the colonization of strain BX-1 were examined every five days for each treatment. The control effects were evaluated after 45 days. After the 15th day of root-knot nematode inoculation, no fluorescently labeled strains were observed in tomato roots and inter-root soil. On the 17th day, some tomato seedlings were kept under observation until the 45th day, while other parts of the tomato seedlings received additional supplementation with the same amount of the strain to continue subsequent experiments.

### 2.5. The Determination of GFP Strain Colonization

For the determination of the GFP strain colonization in tomato roots, we first removed the tomato seedlings and then carefully detached the soil attached to the roots. The roots were subsequently cut into 1 cm long segments. A total of 0.3 g of each plant was thoroughly mixed together, and 0.3 g of the strain was examined under a fluorescence microscope to observe colonization. The remaining portion was placed into a sterile mortar and pestle, where 1.5 mL of sterile water was added before grinding the samples sufficiently. A small amount of this mixture was diluted, coated onto a resistant plate, and incubated overnight to count the number of colonies.

To determine the colonization of GFP strains in tomato inter-root soil, inter-root soil from plants was mixed with tomato root soil to provide a total sample weight of 10 g. Three grams were then placed in a 50 mL triangular flask containing 27 mL of sterile water and shaken for 30 min to suspend the soil adequately. After standing for an additional 10 min, a small amount of the diluted sample was coated onto a resistant plate for overnight incubation before counting the number of colonies present.

### 2.6. Metabolome of Tomato Root Secretions Before and After Meloidogyne incognita Infestation

The root secretion collection involved the use of two treatments: QR, which was inoculated with the root-knot nematode; JK, which was not inoculated with the root-knot nematode. Tomato seedlings were cultured as previously described and removed 45 days later. The soil was washed away to eliminate root-soil and impurities, and infested tomato roots were carefully picked to remove egg masses and then rinsed three times in sterile water. A 1% NaClO solution was used for a 30 min disinfection process, followed by another rinse in sterile water. Each treatment group was randomly divided into three groups of 10 seedlings, which were then placed into triangular vials containing 250 mL of sterile water and sealed with cotton wool. The samples were incubated in a constant temperature light incubator for 48 h [25]. Afterward, the liquid was collected into a 50 mL centrifuge tube, centrifuged at 4500 rpm for 10 min, filtered using a 0.22 µm filter membrane, and stored at −80 °C.

The tomato root secreted sample was extracted with ethyl acetate phase, and the collected sample was dissolved with chromatographic methanol to a concentration of 1 mg mL^−1^. The sample was then filtered with a 0.22 μm filter tip and stored in the injection vials, and this was entrusted to BioMarker Biotechnology, 6th Floor, Shunjie Building, No. 12, Nanfa Xinfu Front Street, Shunyi District, Beijing for UPLC-MS/MS detection and analysis.

The elution gradient was as follows. At 0.00 min, the proportion of B phase was 5%, the proportion of B phase increased linearly to 95% within 9.00 min, and it was then maintained at 95% for 1 min. The proportion of B phase decreased to 5% and was then equilibrated at 5% for 14 min. From 10.00 to 11.10 min, the phase ratio decreased to 5% and was then equilibrated at 5% for 14 min.

Metabolite structure identification was based on the BioMarker Metabolic database(Ultra-high performance liquid phase, Waters UPLC Acquity I-Class PLUS, Waters; High-resolution mass spectrometry, Waters UPLC Xevo G2-XS QTOF Waters; Columns Acquity UPLC HSS T3 1.8 μm 2.1*100 mm, Waters).

### 2.7. Chemotactic Assay

According to the method by Parales et al. [26], the OD600 of the fermentation broth of the strain was adjusted to 0.02, 300 μL was added to a 24-well plate, and 1 μL of the root secretion sample was aspirated in a capillary tube (inner diameter of 0.5 mm, length of 23 mm) and inserted into a 24-well plate. Aqueous solutions of water and 10% methanol were used as controls, respectively, and after 45 min, the liquid in the tube was blown into a 1.5 mL centrifuge tube. The plate was then diluted and coated, and the number of colonies on the plate was recorded statistically 24 h later. Three replicates were performed for each strain, and the experiments were repeated three times. The chemotaxis response of the strains to tomato root secretions was expressed as the chemotaxis index (R), where R = number of colonies in the experimental group/number of colonies in the control group (R > 1: positive chemotaxis) [27].

Differential metabolites in the KEGG database that are abundant, highly variable, and associated with plant systemic resistance metabolic pathways, as well as those found in HMDB and related to microbial chemotaxis activities, were selected for validation of the strain BX-1 chemotaxis. By combining the results of the metabolome analysis and literature search, differential metabolites in metabolic pathways, such as phenylpropanoid biosynthesis, riboflavin biosynthesis, phenylalanine metabolism, ubiquinone and other terpene-quinone biosynthesis, tyrosine metabolism, monoterpene biosynthesis, plant signaling hormones, fatty acids, carboxylic acids and their derivatives, phenolics, and benzene and sterol derivatives, were identified for activity validation based on a significance level of *p* < 0.03 and VIP > 1.3.

### 2.8. Data Analysis

The data were subjected to a one-way analysis of variance (ANOVA) and Tukey’s test using IBM SPSS Statistics 26.0. The error bars are presented as mean (M) ± standard deviation (SD). Significant differences were determined using a *t* test, with * indicating *p* < 0.05, ** indicating *p* < 0.01, and *** indicating *p* < 0.001.

## 3. Results

### 3.1. Nematicidal Activity of Proteus vulgaris BX-1

**Figure 1:**
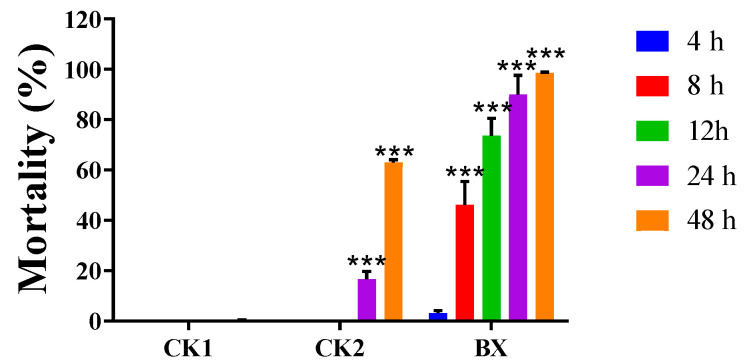
The mortality rate of *Meloidogyne incognita* caused by the fermentation broth of strain BX-1 at various time intervals. CK1: sterile water; CK2: sterile LB liquid medium. BX: Strain BX-1 fermentation broth *** *p* < 0.001.

The fermentation broth of strain BX-1 had a significant virulence effect on *M. incognita*, reaching 73.64% at 12 h, 89.91% at 24 h, and 98.50% at 48 h (Figure 1).

### 3.2. Biocontrol Efficiency of Proteus vulgaris BX-1

**Figure 2:**
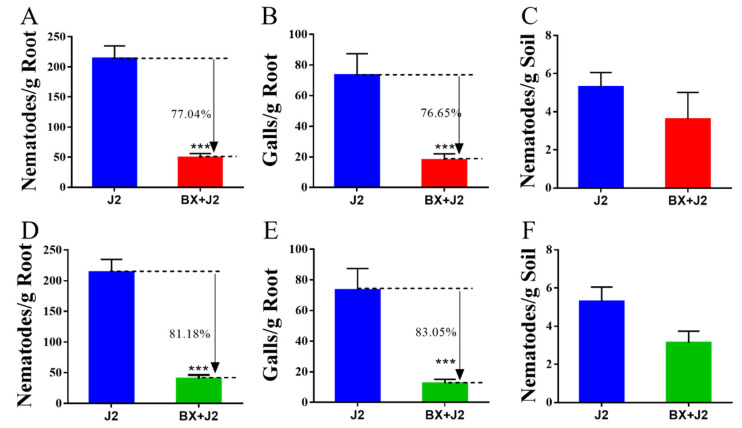
The biocontrol efficiency of strain BX-1: (**A**,**D**) Nematodes in root; (**B**,**E**) Galls of root; (**C**,**F**) Nematodes in root; four treatments were set up in this experiment, with five replicates for each treatment; (**A**–**C**) Apply fermentation broth before inoculating *M. incognita*; (**E**,**F**) Fermentation broth was added after 17 days after inoculation with *M. incognita*. *** *p* < 0.001.

As can be seen from Figure 2, the strain BX-1 fermentation broth treatment was found to significantly inhibit root-knot nematode infestation 45 days after inoculation with *M. incognita*. Compared with the control, the application of the fermentation solution before inoculation with nematodes reduced the number of nematodes in tomato roots by 77.04% and the number of root knots by 76.65%, and its preventive effect was improved by the addition of the fermentation solution at day 17, which reduced the number of nematodes in the roots by 81.18% and the number of root knots by 83.05%. The number of nematodes in the soil did not differ significantly from that of the control Supplementary Tables S2 and S3.

### 3.3. Colonization of Proteus vulgaris BX-1 After Nematode Infestation

**Figure 3:**
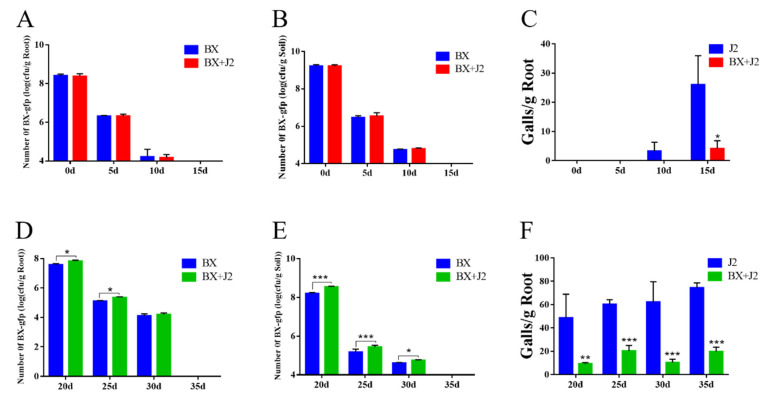
Colonization of strain BX-1: (**A**,**D**) Colonization of strain BX in tomato roots; (**B**,**E**) Colonization of strain BX in tomato rhizosphere soil; (**C**,**F**) Root-knot nematode infestation; (**A**–**C**) Apply fermentation broth before inoculating root-knot nematodes; (**D**–**F**) Inoculated with root-knot nematodes for 17 days. (**A**–**C**) Apply fermentation broth before inoculating *M. incognita*; (**D**–**F**) Fermentation broth was added again after 17 days of inoculation with *M. incognita*. Four treatments were set up in this experiment, with five replicates for each treatment. * *p* < 0.05, ** *p* < 0.01, *** *p* < 0.001.

As depicted in Figure 3, the fluorescently labeled strain BX-1 was observed in the roots of tomato plants and the surrounding soil after approximately 15 days. The colonization of both the tomato roots and inter-root soil by strain BX-1 within 10 days of root-knot nematode inoculation did not show a significant difference compared to that of healthy plants. Additionally, at this time point, no root-knot formation was evident in the treatment group that had been treated with fermentation broth.

Furthermore, the colonization of tomato roots by strain BX-1 was found to be significantly higher than that in healthy plants at days 20, 25, and 30 following root-knot nematode inoculation. Similarly, at these time points (i.e., days 20, 25, and 30), strain BX-1 exhibited significantly higher levels of colonization compared to healthy plants. Notably, at both day 20 and day 25 post-inoculation with root-knot nematodes, the colonization of strain BX-1 in the inter-root soil of tomato plants was also significantly higher than that observed in healthy plants.

It is worth mentioning that by this stage (i.e., day 20 and day 25), root nodules had already formed in the treatment group where the fermentation broth had been applied. This observation suggests a change in chemotaxis for strain BX-1 towards being more inclined to colonize the tomato root system after nodules were produced.

### 3.4. Metabolome Changes of Tomato Root Exudates Infected by Meloidogyne incognita

**Figure 4:**
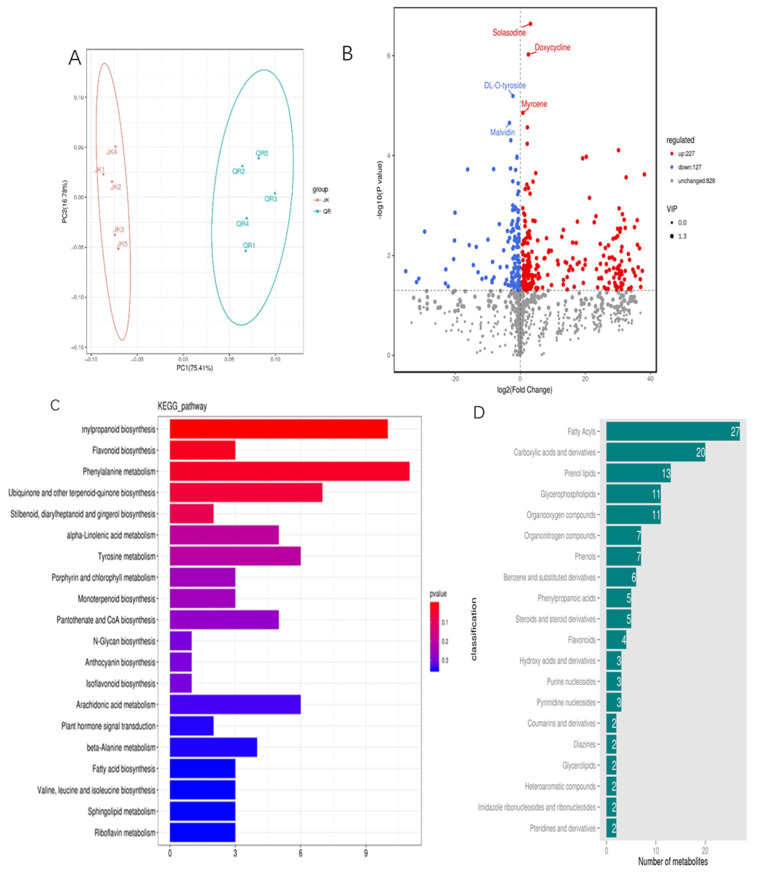
Metabolome analysis of tomato root exudates: (**A**) Principal component analysis; (**B**) Differential metabolite volcano map; (**C**) KEGG classification; (**D**) HDMB classification. JK: exudates of healthy tomato root; QR: exudates of *M. incognita* infested tomato root. In this experiment, two treatments were set up with 10 replicates each. KEGG: Kyoto Encyclopedia of Genes and Genomes; HDMB: Human Metabolome Database.

As depicted in Figure 4, the PCA plot revealed a significant distinction between the treated and control samples, indicating substantial alterations in tomato root metabolites before and after root-knot nematode infestation. A total of 1182 metabolites were identified through LC-MS analysis, with 354 differential metabolites identified from the volcano plot. Among these differentials, 227 were significantly up-regulated while 127 were down-regulated. The KEGG functional annotation of these differential metabolites highlighted several metabolic pathways that experienced notable changes, including the following: phenylpropanoid biosynthesis; riboflavin biosynthesis; phenylalanine metabolism; ubiquinone and other terpene quinone biosynthesis; diphenyl, diarylheptanoid, and curcumin biosynthesis; α-linolenic acid biosynthesis; tyrosine biosynthesis; monoterpene biosynthesis; plant signaling hormones. Furthermore, the HMDB enrichment analysis indicated that fatty acids, carboxylic acids and their derivatives, glycerophospholipids, organic oxygen/nitrogen compounds, phenols, benzene derivatives, and sterols exhibited the most pronounced changes in this study.

Following the analysis of metabolome data and a comprehensive literature review (refer to Tables S2 and S3), eight differentiated metabolites exhibiting significant changes as shown in Table 1 were selected for chemotaxis activity verification.

### 3.5. Chemotactic Activity of Proteus vulgaris BX-1 to Tomato Exudates

**Figure 5:**
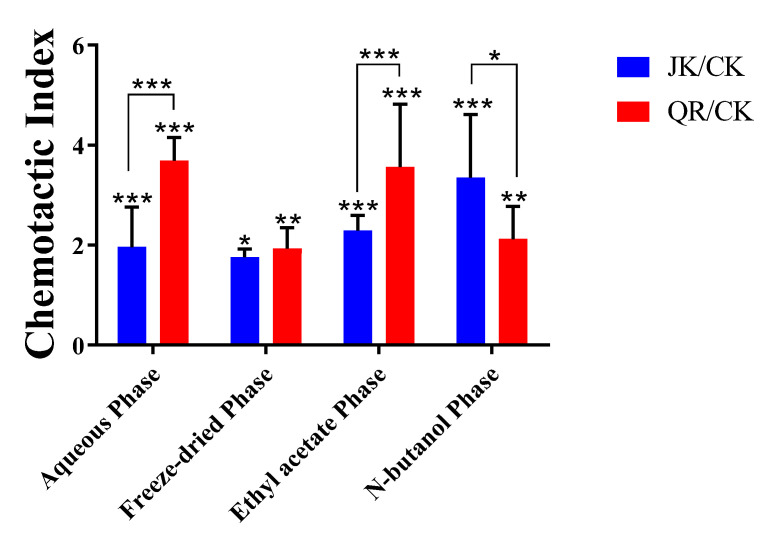
The chemotaxis of strain BX-1 tomato root exudates. CK: sterilized water; JK: exudates of healthy tomato root; QR: exudates of *M. incognita* infested tomato root. Three replicates were performed for each strain, and the experiments were repeated three times. * *p* < 0.05, ** *p* < 0.01, *** *p* < 0.001.

As illustrated in Figure 5, the chemotaxis of strain BX-1 to the aqueous phase samples was significantly enhanced after infestation with root-knot nematodes compared to healthy plants. The active components were predominantly found in the ethyl acetate phase of the root secretions, while the n-butanol phase samples exhibited chemotaxis to BX-1. It is worth noting that strain BX-1 displayed chemotactic behavior towards both healthy and root-knot nematode-infested tomato root secretions.

### 3.6. Chemotaxis Activity of Strain BX-1 to Specific Metabolites

**Figure 6:**
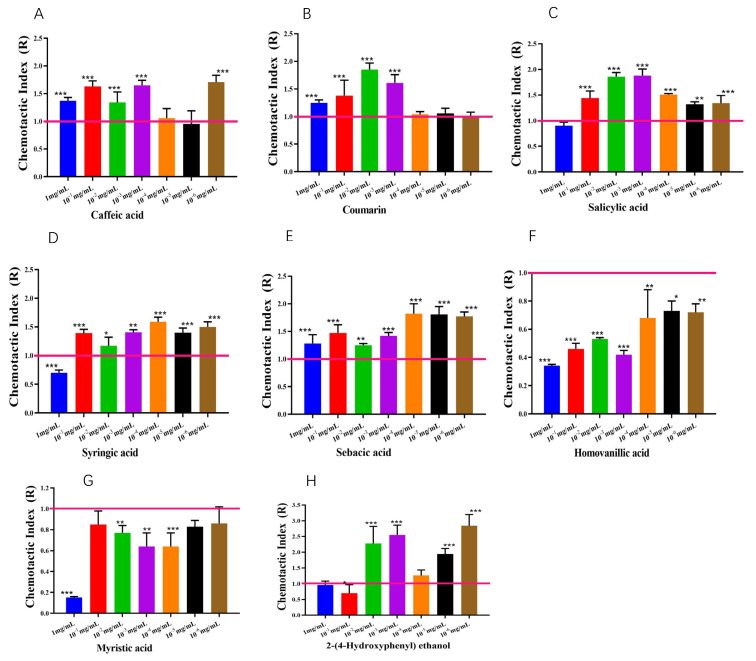
The chemotaxis of strain BX-1 to differential metabolites: (**A**) caffeic acid; (**B**) coumain (**C**) salicylic acid; (**D**) syeingic acid; (**E**) sebacic acid; (**F**) homovanillic acid; (**G**) myristic acid; (**H**) 2-(4-Hydroxyphenyl) ethanol. Three replicates were performed for each strain, and the experiments were repeated three times. * *p* < 0.05, ** *p* < 0.01, *** *p* < 0.001.

As depicted in Figure 6, strain BX-1 exhibited varying chemotaxis towards each compound at different concentrations. In general, it demonstrated significant positive chemo-taxis towards DM-2 (caffeic acid), DM-3 (coumarin), DM-7 (salicylic acid), DM-14 (sebacic acid), and DM-15 (butyric acid) with maximum chemotaxis values of 1.71, 1.85, 1.88, 1.82, and 1.59 corresponding to concentrations of 10^−6^ mg/mL, 10^−2^ mg/mL, 10^−3^ mg/mL and 10^−4^ mg/mL, respectively. Furthermore, it also displayed significant chemotaxis towards DM-8 (homovanillic acid), DM-9 (p-hydroxyphenyl ethanol), and DM-12 (myristic acid) with maximal chemotaxis values of 0.34, 0.46, and 0.15 corresponding to concentrations of 1 mg/mL, 10^−2^ mg/mL, and 1 mg/mL, respectively.

## 4. Discussion

The mortality rate of *M. incognita* treated with *P. vulgaris* BX-1 fermentation broth on the plate reached 98.5% within 48 h. In the pot experiment, the control efficiency reached 76.6%, and this increased to 83.5% after adding the broth at 17 days. These results demonstrate that the strain exhibits strong biocontrol potential.

Several studies have demonstrated that plants are capable of modulating the composition of rhizosphere microorganisms through the secretion of root exudates. This process ultimately promotes plant growth and enhances resistance to various environmental stresses, including drought [28], pests, and diseases [29]. Topalović et al. [30] illustrated that plants under stress from root-knot nematodes that recruit beneficial microbial communities to form aggregates in the inter-roots as a defense mechanism against infestation. Wen et al. [31] observed that *F. spinosum*-susceptible cucumbers tend to increase the secretion of organic acids in order to promote the aggregation of beneficial microorganisms. Additionally, Shi et al. [32] reported that *Artemisia lutea* roots can enhance their growth by promoting specific rhizosphere microbial communities rich in *Bacteroides*, *Probobacterium*, and other proliferating microorganisms. In this study, the metabolome analysis revealed significant changes in tomato root exudates before and after nematode infection. Through tracking, it was observed that during the first 10 days of root-knot nematode infection (with no root-knot production in the treatment group with fermentation broth), there was no significant difference in the colonization amount of strain BX-1 in tomato roots and rhizosphere soil compared to that of healthy plants. However, on the 20–30th days (when all treatments produced root-knots), its colonization in tomato roots and rhizosphere soil was significantly higher than that of healthy plants, indicating that nematode infection increased the colonization rate of strain BX-1. Significant changes in tomato root exudates should be associated with alterations in the colonization of the strain.

In order for biocontrol strains to be effective, they must successfully colonize the rhizosphere of plants [33]. Plant–bacterial interactions are primarily influenced by root exudates [34,35], and studies have demonstrated that the changes induced by adding root exudates affect the chemotaxis of the strains [36]. The chemotoxicity of beneficial bacteria produced by root exudates is essential for successful root colonization [37,38]. de Weert et al. [8] found that biocontrol strains with *cheA* mutations in chemotaxis and mutant strains lacking motility showed reduced colonization at tomato root tips. Additionally, Gao et al. [16] reported that the addition of concentrated watermelon root exudates significantly increased the colonization of *B. amyloliquequemia* TR2 on the surface of watermelon roots. The chemotaxis of strain BX-1, induced by tomato root exudates, exhibited notable changes, resulting in increased tropism. Several compounds demonstrated significant alterations that were associated with the chemotaxis of BX-1, including DM-2 (caffeic acid), DM-3 (coumarin), DM-7 (salicylic acid), DM-14 (sebacic acid), DM-15 (syringic acid), DM-8 (homovanillic acid), DM-9 (p-hydroxyphenylethanol), and DM-12 (myristic acid).

Caffeic acid, coumarin, salicylic acid, syringic acid, and sebacic acid have been found to exhibit strong attraction effects on strain BX-1. In contrast, homovanillic acid, p-hydroxyphenyl ethanol, and myristic acid have shown strong repulsion effects. Previous studies have reported that tomato roots infected with pathogenic bacteria showed increased levels of caffeic acid [39], which inhibits *Fusarium oxysporum* and enhances plant resistance [40]. Akiosk et al. [41] also noted that coumaric acid and caffeic acid can act as allelopathic substances in the rhizosphere with defensive effects. Salicylic acid-mediated systemic resistance plays a crucial role in plant defense against pests and diseases while affecting bacterial chemotaxis. Salicylic acid has been observed to exert an attractive effect on *Ralstonia solanacearum* [42] and can promote the colonization of the PGPR strain *Pseudomonas chlororaphis* (ZSB15-M2) [43]. Additionally, salicylic acid exhibits NA (LC50 of 46 μg/mL) [44], and it also exerted a strong attraction effect on *Proteus vulgaris* BX-1 in this study. Liu et al. [45] found that the addition of myristic acid increased the number of antagonistic rhizosphere microorganisms in eggplants; however, it exerted a significant avoidance effect on BX-1 in our study. Coumarin, sebacic acid, homovanillic ac-id, and p-hydroxyphenylethyl alcohol identified in this study have not been characterized in terms of microbial chemotaxis.

This study suggests that nematode infestation results in modifications to root secretions, leading to changes in specific compounds that enhance the chemotaxis of *P. vulgaris* BX-1. Consequently, this facilitates their colonization between roots and ultimately contributes to the management of root knot disease. Nevertheless, further investigation is imperative to comprehensively comprehend the impact of these compounds on bacterial strains and how they can be effectively harnessed for nematode disease control.

## Figures and Tables

**Table 1:** The selected compounds for chemotaxis activity verification from KEGG and HMDB.

Number	Name	*p* Value	VIP	Regulated	KEGG Pathway Annotation	HMDB
DM-2	Caffeic acid	0.005	1.52	up		
DM-3	Coumarin	0.011	1.31	up		
DM-7	Salicylic acid	0.015	1.37	up	Phenylalanine metabolism; Plant hormone signal transduction	
DM-8	Homovanillic acid	0.04	1.25	up	Tyrosine metabolism	
DM-9	2-(4-Hydroxyphenyl) ethanol	2.73 × 10^−5^	1.65	up		
DM-12	Myristic acid	0.021	1.39	up		Carboxylic acids and derivatives
DM-14	Sebacic acid	0.027	1.35	up		
DM-15	Syringic acid	0.028	1.30	up		

Note: VIP: Variable Importance in Projection.

## Data Availability

Data are unavailable due to privacy restrictions.

## Supplementary Materials

The following supporting information can be downloaded at: https://www.mdpi.com/article/10.3390/microorganisms12112188/s1, Figure S1: Strain BX-1 green fluorescent marker; Table S1: The growth of the plant; Table S2: The selected compounds of chemotaxis activity verification from KEGG; Table S3: The selected compounds of chemotaxis activity verification from HMDB.
