# Supplementary material for: Tomato Root Exudates Infected by Meloidogyne incognita Impact the Colonization of Nematicidal Proteus vulgaris
Source: Microorganisms. 2024 Oct 30;12(11):2188. doi: 10.3390/microorganisms12112188 (PMC11596294; doi:10.3390/microorganisms12112188)
Supplement: Supplementary file 1 [file microorganisms-12-02188-s001.zip › microorganisms-3240790-supplementary.pdf]

Supplementary Materials

1 Strain BX-1 green fluorescent marker

As can be seen from Figure S1, the electrophoresis test of the translator double enzymatic product found a clear band around 750bp, the size of the sfgfp sequence (717bp) Consistent, green fluorescence under the fluorescence microscope indicates that the gfp gene is successfully expressed in the body of the strain BX-1, and after 10 consecutive generations, the fluorescence is still strong, indicating that the plasmid can stabilize the inheritance. Furthermore, the activity and growth of nematodes are not affected by the markings.

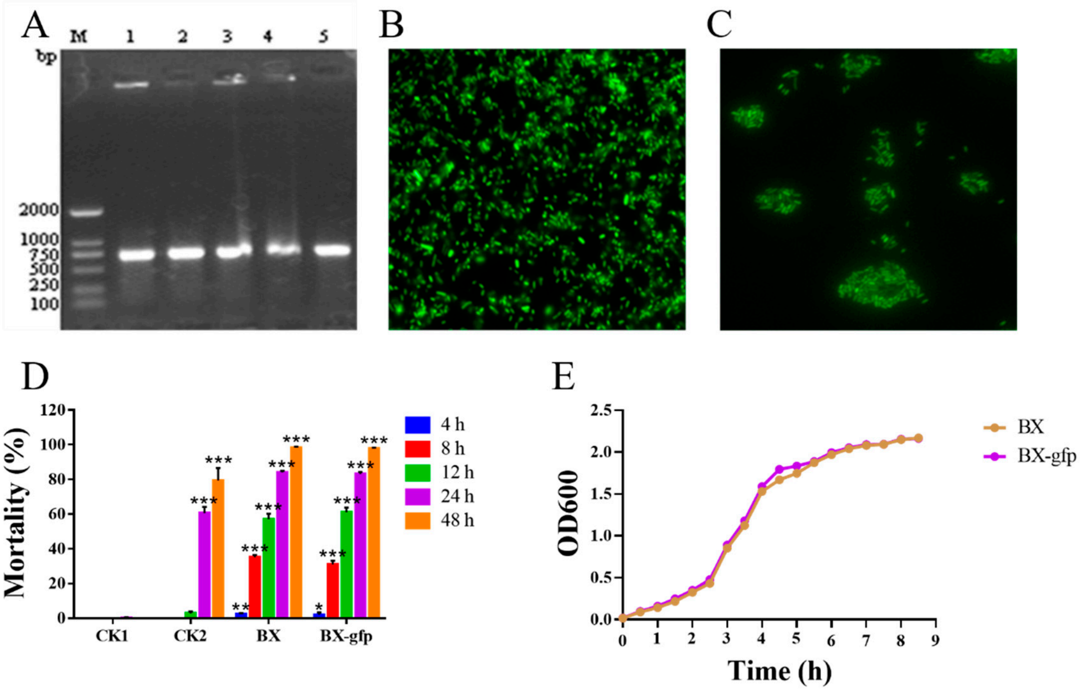

Figure S1. The green fluorescent label of strain BX-1. A: Colony PCR Identification； B: Fluorescent photos； C: Fluorescent photo of plasmid stability；D: Activity of nematode before and after marking；E：Growth curves before and after labeling; * $p<0.05$, ** $p<0.01$, *** $p<0.001$

Table S1. The growth of the plant

Treatment	Shoot Length (cm)	Root Length (cm)	Plant Fresh Weight (g)	Root Fresh Weight (g)
CK	26.17 ±1.27	22.27 ±7.92	7.19 ±1.82	2.51 ±0.27
J2	19.47 ±2.01***	26.50 ±1.32	4.36 ±0.48	2.65 ±0.26
BX-1+J2 (1)	40.73 ±1.37***	16.67 ±1.06	20.42±1.32***	4.25±0.46***
BX-1（1）	42.33 ±1.56***	16.57 ±0.83	21.55 ±0.59***	4.75±0.26***
BX-1+J2 (2)	41.07 ±2.26***	17.03 ±1.99	20.28 ±0.39***	4.85±0.24***
BX-1（2）	46.03 ±1.63***	16.40 ±0.96	22.12 ±1.82***	5.04±0.45***

Note:（1）： Administering strains before inoculation with *Meloidogyne incognita* BX-1；（2）：17 days of inoculation of *Meloidogyne incognita* with additional strains BX-1; * $p<0.05$, ** $p<0.01$, *** $p<0.001$

Table S2. The selected compounds of chemotaxis activity verification from KEGG

Number	Name	P value	VIP	Regulated	KEGG pathway annotation
DM-1	4-Allyl-2-methoxyphenol	0.002	1.60	up	Phenylpropanoid biosynthesis
DM-2	Caffeic acid	0.005	1.52	up	
DM-3	Coumarin	0.011	1.31	up	
DM-4	4-Vinyl phenol	0.044	1.17	up	
DM-5	(-)-Naringenin youpisu	0.043	1.21	up	Soflavonoid biosynthesis
DM-6	trans-Cinnamic acid	0.025	1.31	up	Phenylalanine metabolism; Ubiquinone and other terpenoid-quinone biosynthesis
DM-7	Salicylic acid	0.015	1.37	up	Phenylalanine metabolism; Plant hormone signal transduction
DM-8	Homovanillic acid	0.04	1.25	up	Tyrosine metabolism
DM-9	2-(4-Hydroxyphenyl) ethanol	2.73E	1.65	up	
DM-10	Myrcene	1.40E	1.67	up	Monoterpenoid biosynthesis
DM-11	Menthone	0.047	1.21	up	

Table S3. The selected compounds of chemotaxis activity verification from HMDB

Number	Name	P value	VIP	Regulated	HMDB
DM-12	Myristic acid	0.021	1.39	up	Carboxylic acids and derivalines
DM-13	(2-trans,6-trans)-Farnesol	0.024	1.33	up	
DM-14	Sebacic acid	0.027	1.35	up	
DM-15	Syringic acid	0.028	1.30	up	
DM-16	10-hydroxy capric acid	0.019	1.40	up	
DM-17	Embelin	0.029	1.32	up	Phenols

Note: VIP: Variable Importance in Projection. E： multiply 10^{-5}
